# Case Report: Myxedema Coma Caused by Immunoglobulin A Vasculitis in a Patient With Severe Hypothyroidism

**DOI:** 10.3389/fimmu.2022.838739

**Published:** 2022-02-18

**Authors:** Hiroshi Ito, Kenzo Fukuda, Kenji Ashida, Ayako Nagayama, Tomoki Sako, Kouichiro Mizuochi, Masaharu Kabashima, Satoko Yoshinobu, Shimpei Iwata, Nao Hasuzawa, Sumika Hayashi, Tomoyuki Akashi, Masatoshi Nomura

**Affiliations:** ^1^ Division of Endocrinology and Metabolism, Department of Internal Medicine, Kurume University School of Medicine, Kurume, Japan; ^2^ Department of Diabetology, Shimada Hospital, Ogori, Japan; ^3^ Department of Intensive Care Medicine, Imamura Hospital, Tosu, Japan

**Keywords:** glucocorticoid, Hashimoto’s thyroiditis, IgA vasculitis, levothyroxine, liothyronine, myxedema coma

## Abstract

Myxedema coma is a critical disorder with high mortality rates. Disruption of the compensatory mechanism for severe and long-term hypothyroidism by various causes leads to critical conditions, including hypothermia, respiratory failure, circulatory failure, and central nervous system dysfunction. Infectious diseases, stroke, myocardial infarction, sedative drugs, and cold exposure are considered the main triggers for myxedema coma. A 59-year-old Japanese woman presented with bilateral painful purpura on her lower legs. She was diagnosed with coexisting immunoglobulin A (IgA) vasculitis and severe IgA vasculitis with nephritis and was consequently treated with intravenous methylprednisolone (125 mg/day). However, she rapidly developed multiple organ failure due to the exacerbation of severe hypothyroidism, i.e., myxedema. Her condition improved significantly following oral administration of prednisolone along with thyroxine. There was a delayed increase in the serum free triiodothyronine level, while the serum free thyroxine level was quickly restored to normal. Rapid deterioration of the patient’s condition after admission led us to diagnose her as having myxedema coma triggered by IgA vasculitis. Hence, clinicians should be aware of the risks of dynamic exacerbations in patients with hypothyroidism. Furthermore, our study suggested that combination therapy with thyroxine and liothyronine might prove effective for patients with myxedema coma, especially for those who require high-dose glucocorticoid administration.

## Introduction

Myxedema coma is an endocrine emergency and fatal disease that is rarely encountered (1). Annually, 1.08 cases per million people in Japan and 0.22 cases per million people in Spain develop myxedema coma (2, 3). It can lead to highly critical conditions, such as central nervous system dysfunction, hypothermia, respiratory failure, and circulatory failure (3). Thus, although the incidence of myxedema coma is low, early diagnosis and avoidance of overlooking this disease are required for successful treatment (2).

Severe and long-term hypothyroidism reduces intracellular triiodothyronine (T3) levels, thus leading to lessened sensitivity to high carbon dioxide and low oxygen concentrations, decreased thermogenesis, diminished cardiac output, and increased fluid retention. While compensatory mechanisms maintain homeostasis, various factors—including infectious diseases, stroke, congestive heart failure, sedative drugs, and cold exposure [**Supplementary Table**]—break down this stability and trigger the development of myxedema coma (4–8). Respiratory failure (due to hypoventilation), hypothermia, circulatory failure, and central nervous system dysfunction are considered the major clinical symptoms of myxedema coma.

Although the pathogenic mechanism of immunoglobulin A (IgA) vasculitis has yet to be elucidated, it has been suggested that immune complexes deposit mainly on arterial walls and activate the complement system (9). Blood vessel wall destruction by neutrophils causes IgA vasculitis with nephritis (10), the symptoms of which include tactile purpura on the lower legs, arthritis, abdominal pain, and nephropathy. Systemic inflammation caused by autoimmune mechanisms may cause severe hypothyroidism resulting in the development of myxedema coma by disrupting the compensatory mechanism for downregulated T3 expression. In addition, proteinuria related to IgA vasculitis with nephritis may contribute to thyroid hormone deficiency (11–13). However, whether IgA vasculitis causes deterioration of hypothyroidism into myxedema coma requires further clarification. In addition, it remains unclear whether liothyronine (LT3) should be administered in addition to levothyroxine (LT4) to treat myxedema coma, although the efficacy of combination therapy with LT3 and LT4 has been demonstrated previously (14–16).

Here, we present a case of myxedema coma triggered by IgA vasculitis. Dynamic exacerbations of the patient’s condition led us to establish the diagnosis of myxedema coma. Furthermore, T3 depression was highlighted in the clinical features of this case that was treated using glucocorticoids at a pharmacological dose. Clinicians should be aware of myxedema coma as a critical disorder that can cause rapid deterioration.

## Case Description

**Figure 1:**
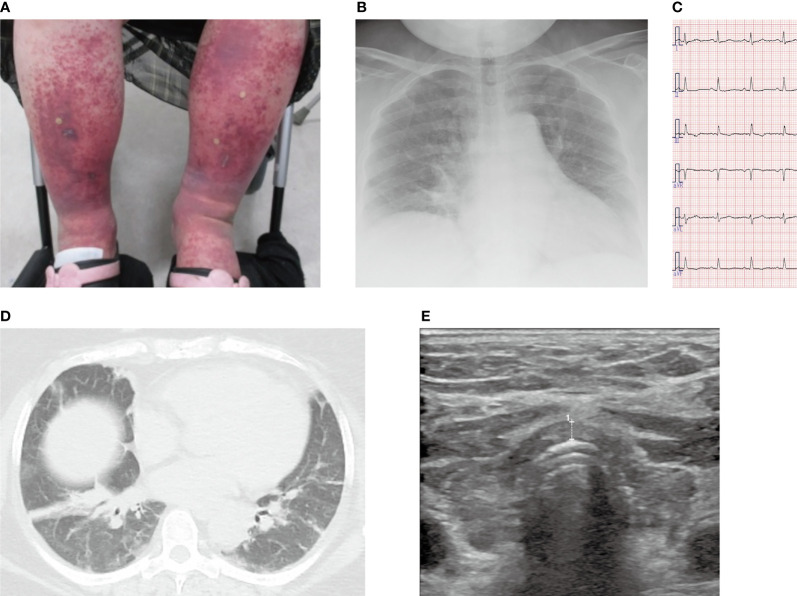
Images of the lower legs, chest X-ray, chest computed tomography, electrocardiogram, and echocardiogram. **(A)** Purpura and edema on both lower legs. **(B)** Chest X-ray on admission showed mild pulmonary congestion. **(C)** No abnormalities were found on the electrocardiogram at the time of admission. **(D)** Chest computed tomography showed atelectasis and mild pulmonary congestion. **(E)** Thyroid echo showed diffuse atrophy of the thyroid gland, irregular surface, and rough and low echo levels inside the thyroid gland. The thickness of the isthmus is 2.6 mm in diameter, which is indicated as broken line 1.

A 59-year-old Japanese woman visited a general outpatient center of Shimada Hospital, Fukuoka, Japan, complaining of purpura on both her lower legs and difficulty in walking because of ankle pain. She had no medical history other than obesity. On physical examination, her consciousness was clear. Her height, body weight, and body mass index were 160.0 cm, 113.0 kg, and 44.1 kg/m^2^, respectively. She had a blood pressure of 101/63 mmHg, a pulse rate of 66 beats/min, a body temperature of 36.5°C, and an oxygen saturation of 93% (room air). Her eyelids, face, and extremities were edematous; however, no goiter was observed. Palpable purpura was found on her abdomen and bilateral lower legs (**Figure 1A**). Additionally, she complained of tenderness and exercise pain in her ankles.

**Figure 2:**
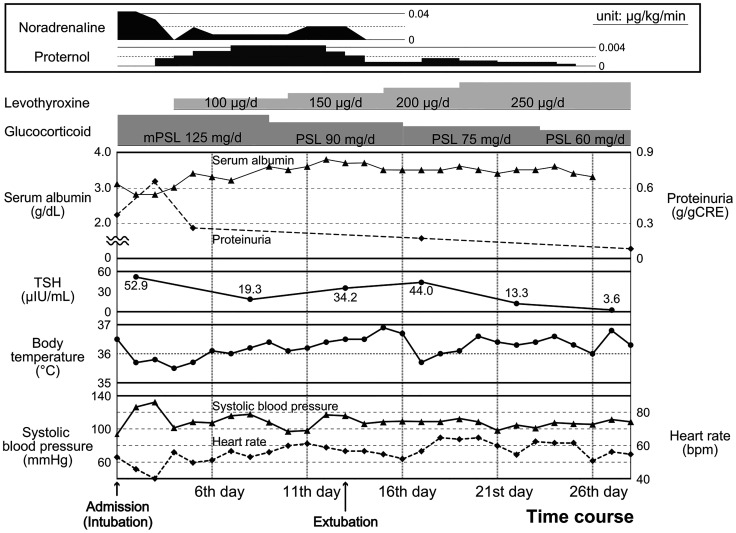
Clinical course after admission. Physical findings: body temperature (closed circles with solid lines), systolic blood pressure (closed triangles with solid lines), and heart rate (square with broken lines) [lower]; laboratory findings: serum albumin (closed triangles with solid lines), proteinuria (square with broken lines), and TSH levels (closed circles with solid lines) [middle]; and the contents of medication therapies [upper] are presented.

Blood tests disclosed renal failure (high serum creatinine levels) and elevated serum C-reactive protein levels, and arterial blood gas analysis showed type II respiratory failure (**Table 1**). Chest X-ray demonstrated enlargement of the cardiothoracic ratio by 61.0% (**Figure 1B**). Electrocardiography indicated no abnormalities (**Figure 1C**), whereas echocardiography revealed left ventricular diastolic dysfunction; however, no abnormal contractility or pericardial fluid retention was detected. Atelectasis and mild pulmonary congestion were observed on computed tomography (**Figure 1D**). Furthermore, hypothyroidism (positive for antithyroid antibodies) and a diffusely atrophic thyroid gland (visualized by ultrasonography) were suggestive of Hashimoto’s thyroiditis (**Figure 1E**). A pathological examination of purpura revealed vasculitis-compatible features: perivascular infiltration and nuclear fragmentation of inflammatory cells. Thus, she was diagnosed with coexisting IgA vasculitis, purpura in the lower legs and abdominal skin, bilateral ankle arthritis, and nephropathy. Diagnosis of severe IgA vasculitis with nephritis, indicated by high serum creatinine level, proteinuria, and hematuria (**Table 1**, **Figure 2** and **Supplementary Figure**) led us to administer glucocorticoids at a pharmacological dose of methylprednisolone (125 mg/day) intravenously.

**Table 1:** Laboratory data on admission.

Parameters	Values	Reference range	Parameters	Values	Reference range
**Complete blood cell count**			**Endocrinology**		
WBC, cells/µL	7,600	3,300–8,600	TSH, µIU/mL	*52.9*	0.5–5.0
Neutrophil, %	*91.5*	40–74	Free thyronine, pg/mL	*0.81*	2.3–4.3
Eosinophil, %	2.6	0–6	Free thyroxine, ng/dL	*0.14*	0.9–1.7
Lymphocyte, %	*3.8*	18–59	ACTH, pg/mL	22.2	7.2–63.3
Monocyte, %	2.1	0–8	Cortisol, µg/dL	*22.0*	6.2–19.4
RBC count, cells×10^4^/µL	*324*	386–492	HbA1C, % (NGSP)	*6.4*	4.9–6.0
Hemoglobin, g/dL	*10.5*	11.6–14.8			
Hematocrit, %	*32.1*	35.1–44.4	**Immunology**		
Mean corpuscular volume, fL	*99.1*	83.6–98.2	C-reactive protein, mg/dL	*23.2*	0–0.14
Platelet count, cells×10^4^/µL	*13.1*	15.8–34.8	Anti-TPO Ab, IU/mL	*422*	<16
			Anti-Tg Ab, IU/mL	*3,590*	<28
**Serum chemistry**			Antinuclear Ab, times	<40	<40
Total protein, g/dL	7.8	6.6–8.1	Anti-dsDNA Ab, IU/mL	<10	0–12
Albumin, g/dL	*3.1*	4.1–5.1	Anticardiolipin Ab, U/mL	<8	0–9.9
AST, IU/L	21	13–30	PR3-ANCA, U/mL	<1.0	<3.5
ALT, IU/L	*27*	7–23	MPO-ANCA, U/mL	<1.0	<3.5
Lactate dehydrogenase, IU/L	*226*	124–222	Anti-SSA Ab, U/mL	<1.0	0–10
Alkaline phosphatase, IU/L	147	106–322	IgA, mg/dL	132	110–410
Blood urea nitrogen, mg/dL	*42.3*	8–20	HBs antigen	Negative	
Creatinine, mg/dL	*2.18*	0.46–0.79	HCV Ab	Negative	
Sodium, mmol/L	137	138–145	CH_50,_ U/m	*55.2*	25–48
Potassium, mmol/L	4.0	3.6–4.8	C_3_, mg/dL	*172*	86–160
Chloride, mmol/L	*96*	101–108	C_4_, mg/dL	35	17–45
Total cholesterol, mg/dL	193	142–248			
FPG, mg/dL	104	73–109	**Coagulation**		
BNP, pg/mL	*54.3*	0–18.4	PT/INR	1.01	
			APTT, second	29.0	23–36
**Blood gas analysis (O_2_: 3 L/min)**			D-dimer, µg/mL	*4.7*	0–1.0
pH	7.37	7.35–7.45			
PaO_2_, mm Hg	*78.1*	85–95	**Urinalysis**		
PaCO_2_, mm Hg	*53.3*	36.4–44.4	Protein, g/gCRE	*0.66*	
$\text{HC}{\text{O}}_{3}^{-}$ , mmol/L	*29.5*	22–26	RBC count/HPF	*20–29*	<1
Lactate, mg/dL	7.7	4–16			
PaO_2_/FiO_2_ ratio	*244*				

Abnormal values are shown in italics. Ab, antibody; ACTH, adrenocorticotropic hormone; ALT, alanine aminotransferase; APTT, activated partial thromboplastin time; AST, aspartate aminotransaminase; BNP, brain natriuretic peptide; dsDNA, double-stranded DNA; FiO_2_, fraction of inspired oxygen; FPG, fasting plasma glucose; HbA1c, Hemoglobin A1C; HBs, hepatitis B surface; HCV, hepatitis C virus; HPF, high-power field; IgA, immunoglobulin A; MPO-ANCA, myeloperoxidase–antineutrophil cytoplasmic antibody; PaO_2_, partial pressure of arterial oxygen; PaCO_2_, partial pressure of arterial carbon dioxide; PR3-ANCA, proteinase 3-antineutrophil cytoplasmic antibody; PT/INR, prothrombin time/international normalized ratio; RBC, red blood cell count; SSA, Sjögren’s syndrome A; Tg, Thyroglobulin; TPO, Thyroid peroxidase; TSH, Thyroid-stimulating hormone; WBC, white blood cell count.

A few hours after admission, the patient developed impaired consciousness, respiratory failure, and circulatory failure. Hypoxemia and hypoventilation necessitated forced ventilation by a respirator. Moreover, noradrenaline, dopamine, and isoprenaline were administered to treat her circulatory failure along with bradycardia (heart rate < 45 beats/min) and shock. Impaired consciousness, hypothermia (35.5°C), bradycardia, type II respiratory failure, and hypothyroidism identified on the fourth day of admission led to the diagnosis of myxedema coma based on the diagnostic scoring system for myxedema coma (17). Thyroid hormone replacement therapy with LT4 and without LT3 was immediately started at an initial dose of 100 μg per day through a nasogastric tube. The clinical course of this case is shown in **Figure 2**.

Intensive treatment in the intensive care unit with prednisolone and LT4 (finally adjusted to 250 µg/day) successfully improved the patient’s condition and recovered thyroid functions; however, she was extubated and discharged from the intensive care unit after 13 and 26 days, respectively (**Figure 2**). As for IgA vasculitis with nephritis, glucocorticoid administration had successfully reduced proteinuria, with peak elevation observed at 0.66 g/gCr on day 3, which reduced to 0.26 g/gCr on day 5, and was finally absent by day 10. In addition, with the reduction in proteinuria and amelioration of systemic inflammation, the serum albumin level showed improvement from 2.8 mg/dL (on days 2 and 3) to 3.4 mg/dL (on day 5), which eventually reached 3.6 mg/dL (on day 10) and was maintained at steady levels (**Figure 2** and **Supplementary Figure**). After prednisolone was tapered to 60 mg/day, she was transferred to another hospital to undergo cholecystectomy for cholecystitis. After 1 year, she regularly visits our hospital for follow-up and medical treatment.

## Discussion

We described the first case of myxedema coma triggered by IgA vasculitis. Rapid deterioration of the patient’s condition after admission led to the diagnosis of myxedema coma. LT3 administration should be considered as an alternative treatment for myxedema coma patients requiring concomitant glucocorticoid administration.

Herein, we presented the first case of myxedema coma that resulted from the worsening of hypothyroidism and subsequently exacerbated by IgA vasculitis. Severe hypothyroidism results in a decreased intracellular T3 concentration, which in turn causes respiratory insufficiency, hypotension, and hyponatremia (18, 19). Physiological compensatory mechanisms preventing from deterioration into critical conditions contribute to the maintenance of homeostasis. However, stressors increase the demand for thyroid hormone synthesis, thus leading to advanced thyroid hormone dysfunction, followed by respiratory failure, hypothermia, circulatory failure, hyponatremia, and neurological dysfunction (i.e., myxedema coma) (6). The systemic inflammation caused by IgA vasculitis may have promoted the secretion of cytokines, such as tumor necrosis factor-α, interleukin (IL)-2, IL-6, and IL-8 (20, 21). This finding was indicated by the high C-reactive protein levels in our patient. The release of cytokines may have suppressed catabolism and energy expenditure, resulting in a decrease and increase in T3 and rT3 levels, respectively (22, 23). This resulted in a decrease in cardiac output due to restricted cardiac contraction and decreased heart rate, which is presumed to have led to myxedema coma caused by worsening circulatory failure (24). However, detailed pathological mechanisms of inflammation, including IgA vasculitis, associated with the development of myxedema coma have not been clarified. In this context, this study revealed that autoinflammation can potentially result in the deterioration of hypothyroidism into myxedema coma.

Proteinuria should be considered a risk factor for exacerbated hypothyroidism. IgA vasculitis with nephritis associated with renal failure can be another plausible reason for the development of myxedema coma. Hypoalbuminemia likely concurrent with proteinuria (**Figure 2**) was suggested to contribute to a decrease in serum thyroid hormone and thyroid-binding globulin levels (11, 12), leading to inadequate supplies of thyroid hormones to the heart, liver, brain, and various organs in a systemic manner (13). Based on the chronic hypothyroidism from Hashimoto’s thyroiditis, loss of thyroid hormone triggered by IgA vasculitis with nephritis may have contributed to myxedema coma.

Rapid aggravation of the patient’s condition after admission led us to diagnose her with myxedema coma. Myxedema coma is an endocrine emergency with a high fatality rate that necessitates thyroid preparations as soon as possible (2). Nevertheless, because myxedema coma occurs suddenly, it is necessary to grasp the dynamic pathological condition during the course. Furthermore, delays resulting from failure to diagnose or wait for confirmation by blood tests have contributed to the high mortality of this disease (7). In fact, the present patient walked in our hospital, complaining of IgA purpura without myxedema coma; yet, she developed myxedema coma within 6 hours. Therefore, clinicians should be aware of dynamic changes in the condition of patients with hypothyroidism. Disturbed consciousness, severe respiratory failure, and heart failure have been reported as the keys to the diagnosis of myxedema coma (17).

**Figure 3:**
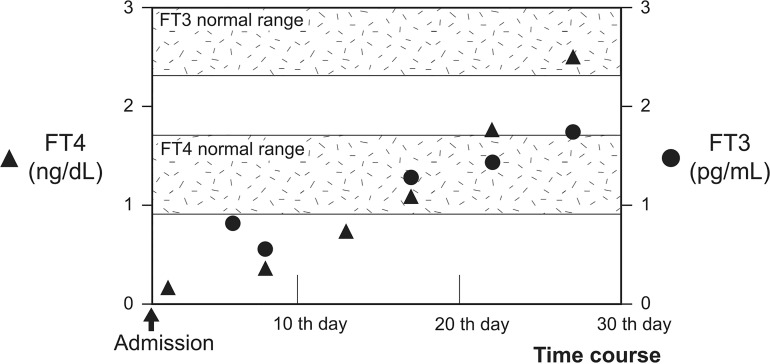
Progress of FT3 and FT4 during hospitalization. Time course of serum FT4 and FT3 levels are shown. Serum FT4 and FT3 levels are denoted by closed triangles and circles, respectively. FT4 levels increased and reached above the normal range following levothyroxine administration after 20th day. However, FT3 levels did not increase sufficiently. The shadows represent the normal ranges of serum free T4 and free T3 levels.

T3 administration should be considered an alternative treatment for patients with myxedema coma who require concomitant glucocorticoid administration. Myxedema coma is often treated with LT4 alone, as recommended by the guidelines in the United States (25) and Latin America (26). Although combination therapy with LT3 and LT4 is not common (14–16), it has reportedly been effective in improving the prognosis in certain cases of myxedema coma (27). Additionally, rapid thyroid hormone replacement is generally avoided because it carries the risk of inducing myocardial infarction and arrhythmias (28). In this context, administration of LT3 was hesitated; however, recovery of serum free T3 levels were delayed in comparison with normal to high levels of serum free T4 levels (**Figure 3**) (16). Moreover, conversion from T4 to T3 has been suggested to be suppressed in myxedema coma (6), especially in cases where pharmaceutical levels of glucocorticoids—which suppress the conversion of T4 to T3 (29, 30) as well as thyroid-stimulating hormone secretion (31)—are administered. The potential for exacerbation of hypothyroidism in response to a pharmacological dose of glucocorticoid should also be noted. Combination therapy with both LT4 and LT3 may prove effective, especially for myxedema coma patients with diseases requiring glucocorticoid administration.

We encountered a patient with myxedema coma triggered by IgA vasculitis. Clinicians should be aware of this critical condition that would be masked by the trigger disorders. Combination therapy with LT4 and LT3 may be useful for patients with myxedema come who require supra-physiological glucocorticoid administration. Further investigation is needed to confirm our conclusions.

## Data Availability Statement

The original contributions presented in the study are included in the article/**Supplementary Material**. Further inquiries can be directed to the corresponding author.

## Ethics Statement

The studies involving human participants were reviewed and approved by Shimada Hospital. The patients/participants provided their written informed consent to participate in this study. Written informed consent was obtained from the individual(s) for the publication of any potentially identifiable images or data included in this article.

## Author Contributions

HI and KA designed the study and drafted the first manuscript. HI collected the data. HI, KF, KA, SH, AN, TS, KM, MK, SY, SI, NH, SH, TA, and MN interpreted the data and provided input in the preparation of the manuscript. HI and KA revised the manuscript. All authors have read and approved the final version of the manuscript.

## Conflict of Interest

The authors declare that the research was conducted in the absence of any commercial or financial relationships that could be construed as a potential conflict of interest.

## Publisher’s Note

All claims expressed in this article are solely those of the authors and do not necessarily represent those of their affiliated organizations, or those of the publisher, the editors and the reviewers. Any product that may be evaluated in this article, or claim that may be made by its manufacturer, is not guaranteed or endorsed by the publisher.

## References

[1] Beynon J Akhtar S Kearney T. Predictors of Outcome in Myxoedema Coma. Crit Care (2008) 12:111. doi: 10.1186/cc6218 18254932 PMC2374614

[2] Ono Y Ono S Yasunaga H Matsui H Fushimi K Tanaka Y. Clinical Characteristics and Outcomes of Myxedema Coma: Analysis of a National Inpatient Database in Japan. J Epidemiol (2017) 27:117–22. doi: 10.1016/j.je.2016.04.002 PMC5350620 28142035

[3] Rodríguez I Fluiters E Pérez-Méndez LF Luna R Páramo C García-Mayor RV. Factors Associated With Mortality of Patients With Myxoedema Coma: Prospective Study in 11 Cases Treated in a Single Institution. J Endocrinol (2004) 180:347–50. doi: 10.1677/joe.0.1800347 14765987

[4] Klubo-Gwiezdzinska J Wartofsky L. Thyroid Emergencies. Med Clin North Am (2012) 96:385–403. doi: 10.1016/j.mcna.2012.01.015 22443982

[5] Ishii M. Endocrine Emergencies With Neurologic Manifestations. Continuum (Minneap Minn) (2017) 23:778–801. doi: 10.1212/CON.0000000000000467 28570329 PMC5902332

[6] Wall CR. Myxedema Coma: Diagnosis and Treatment. Am Fam Physician (2000) 62:2485–90. 11130234

[7] Ylli D Klubo-Gwiezdzinska J Wartofsky L. Thyroid Emergencies. Pol Arch Intern Med (2019) 129:526–34. doi: 10.20452/pamw.14876 PMC6721612 31237256

[8] Spyridoulias A Riaz MS. Myxoedema Coma in the Setting of Hyperglycaemic Hyperosmolar State. BMJ Case Rep (2016) 2016:bcr2015213411. doi: 10.1136/bcr-2015-213411 PMC4716335 26759401

[9] Rigante D Castellazzi L Bosco A Esposito S. Is There a Crossroad Between Infections, Genetics, and Henoch–Schönlein Purpura? Autoimmun Rev (2013) 12:1016–21. doi: 10.1016/j.autrev.2013.04.003 23684700

[10] Heineke MH Ballering AV Jamin A Ben Mkaddem S Monteiro RC Van Egmond M. New Insights in the Pathogenesis of Immunoglobulin A Vasculitis (Henoch-Schönlein Purpura). Autoimmun Rev (2017) 16:1246–53. doi: 10.1016/j.autrev.2017.10.009 29037908

[11] Ito S Kano K Ando T Ichimura T. Thyroid Function in Children With Nephrotic Syndrome. Pediatr Nephrol (1994) 8:412–5. doi: 10.1007/BF00856516 7947028

[12] Benvenga S Vita R Bari FD Fallahi P Antonelli A. Do Not Forget Nephrotic Syndrome as a Cause of Increased Requirement of Levothyroxine Replacement Therapy. Eur Thyroid J (2015) 4:138–42. doi: 10.1159/000381310 PMC4521056 26280000

[13] Santoro D Vadalà C Siligato R Buemi M Benvenga S. Autoimmune Thyroiditis and Glomerulopathies. Front Endocrinol (Lausanne) (2017) 8:119. doi: 10.3389/fendo.2017.00119 28626447 PMC5454061

[14] Shakir MKM Brooks DI McAninch EA Fonseca TL Mai VQM Bianco AC. Comparative Effectiveness of Levothyroxine, Desiccated Thyroid Extract, and Levothyroxine+Liothyronine in Hypothyroidism. J Clin Endocrinol Metab (2021) 106:e4400–13. doi: 10.1210/clinem/dgab478 PMC8530721 34185829

[15] Taylor PN Eligar V Muller I Scholz A Dayan C Okosieme O. Combination Thyroid Hormone Replacement; Knowns and Unknowns. Front Endocrinol (Lausanne) (2019) 10:706. doi: 10.3389/fendo.2019.00706 31695677 PMC6817486

[16] Ettleson MD Bianco AC. Individualized Therapy for Hypothyroidism: Is T4 Enough for Everyone? J Clin Endocrinol Metab (2020) 105:e3090–104. doi: 10.1210/clinem/dgaa430 PMC7382053 32614450

[17] Popoveniuc G Chandra T Sud A Sharma M Blackman MR Burman KD. A Diagnostic Scoring System for Myxedema Coma. Endocr Pract (2014) 20:808–17. doi: 10.4158/EP13460.OR 24518183

[18] Klein I Danzi S. Thyroid Disease and the Heart. Circulation (2007) 116:1725–35. doi: 10.1161/CIRCULATIONAHA.106.678326 17923583

[19] Chaker L Bianco AC Jonklaas J Peeters RP. Hypothyroidism. Lancet (2017) 390:1550–62. doi: 10.1016/S0140-6736(17)30703-1 PMC6619426 28336049

[20] Del Vecchio GC Penza R Altomare M Piacente L Aceto G Lassandro G. Cytokine Pattern and Endothelium Damage Markers in Henoch-Schönlein Purpura. Immunopharmacol Immunotoxicol (2008) 30:623–9. doi: 10.1080/08923970801973646 18668398

[21] Jen H-Y Chuang Y-H Lin S-H Chiang B-L Yang Y-H. Increased Serum Interleukin-17 and Peripheral Th17 Cells in Children With Acute Henoch-Schönlein Purpura. Pediatr Allergy Immunol (2011) 22:862–8. doi: 10.1111/j.1399-3038.2011.01198.x 21929599

[22] Fliers E Bianco AC Langouche L Boelen A. Thyroid Function in Critically Ill Patients. Lancet Diabetes Endocrinol (2015) 3:816–25. doi: 10.1016/S2213-8587(15)00225-9 PMC4979220 26071885

[23] Quispe E Á Li X-M Yi H. Comparison and Relationship of Thyroid Hormones, IL-6, IL-10 and Albumin as Mortality Predictors in Case-Mix Critically Ill Patients. Cytokine (2016) 81:94–100. doi: 10.1016/j.cyto.2016.03.004 26974766

[24] Majid-Moosa A Schussler JM Mora A. Myxedema Coma With Cardiac Tamponade and Severe Cardiomyopathy. Proc (Bayl Univ Med Cent) (2015) 28:509–11. doi: 10.1080/08998280.2015.11929326 PMC4569241 26424958

[25] Jonklaas J Bianco AC Bauer AJ Burman KD Cappola AR Celi FS. Guidelines for the Treatment of Hypothyroidism: Prepared by the American Thyroid Association Task Force on Thyroid Hormone Replacement. Thyroid (2014) 24:1670–751. doi: 10.1089/thy.2014.0028 PMC4267409 25266247

[26] Brenta G Vaisman M Sgarbi JA Bergoglio LM Andrada NC Bravo PP. Clinical Practice Guidelines for the Management of Hypothyroidism. Arq Bras Endocrinol Metabol (2013) 57:265–91. doi: 10.1590/S0004-27302013000400003 23828433

[27] Ueda K Kiyota A Tsuchida M Okazaki M Ozaki N. Successful Treatment of Myxedema Coma With a Combination of Levothyroxine and Liothyronine. Endocr J (2019) 66:469–74. doi: 10.1507/endocrj.EJ18-0469 30853666

[28] Fliers E Wiersinga WM. Myxedema Coma. Rev Endocr Metab Disord (2003) 4:137–41. doi: 10.1023/A:1022985902253 12766541

[29] LoPresti JS Eigen A Kaptein E Anderson KP Spencer CA Nicoloff JT. Alterations in 3,3'5'-Triiodothyronine Metabolism in Response to Propylthiouracil, Dexamethasone, and Thyroxine Administration in Man. J Clin Invest (1989) 84:1650–6. doi: 10.1172/JCI114343 PMC304032 2808705

[30] Carroll R Matfin G. Endocrine and Metabolic Emergencies: Thyroid Storm. Ther Adv Endocrinol Metab (2010) 1:139–45. doi: 10.1177/2042018810382481 PMC3475282 23148158

[31] Brabant G Brabant A Ranft U Ocran K Köhrle J Hesch RD. Circadian and Pulsatile Thyrotropin Secretion in Euthyroid Man Under the Influence of Thyroid Hormone and Glucocorticoid Administration. J Clin Endocrinol Metab (1987) 65:83–8. doi: 10.1210/jcem-65-1-83 3584402

