# Supplementary figures and images for: Case Report: Myxedema Coma Caused by Immunoglobulin A Vasculitis in a Patient With Severe Hypothyroidism
Source: Front Immunol. 2022 Feb 18;13:838739. doi: 10.3389/fimmu.2022.838739 (PMC8895252; doi:10.3389/fimmu.2022.838739)

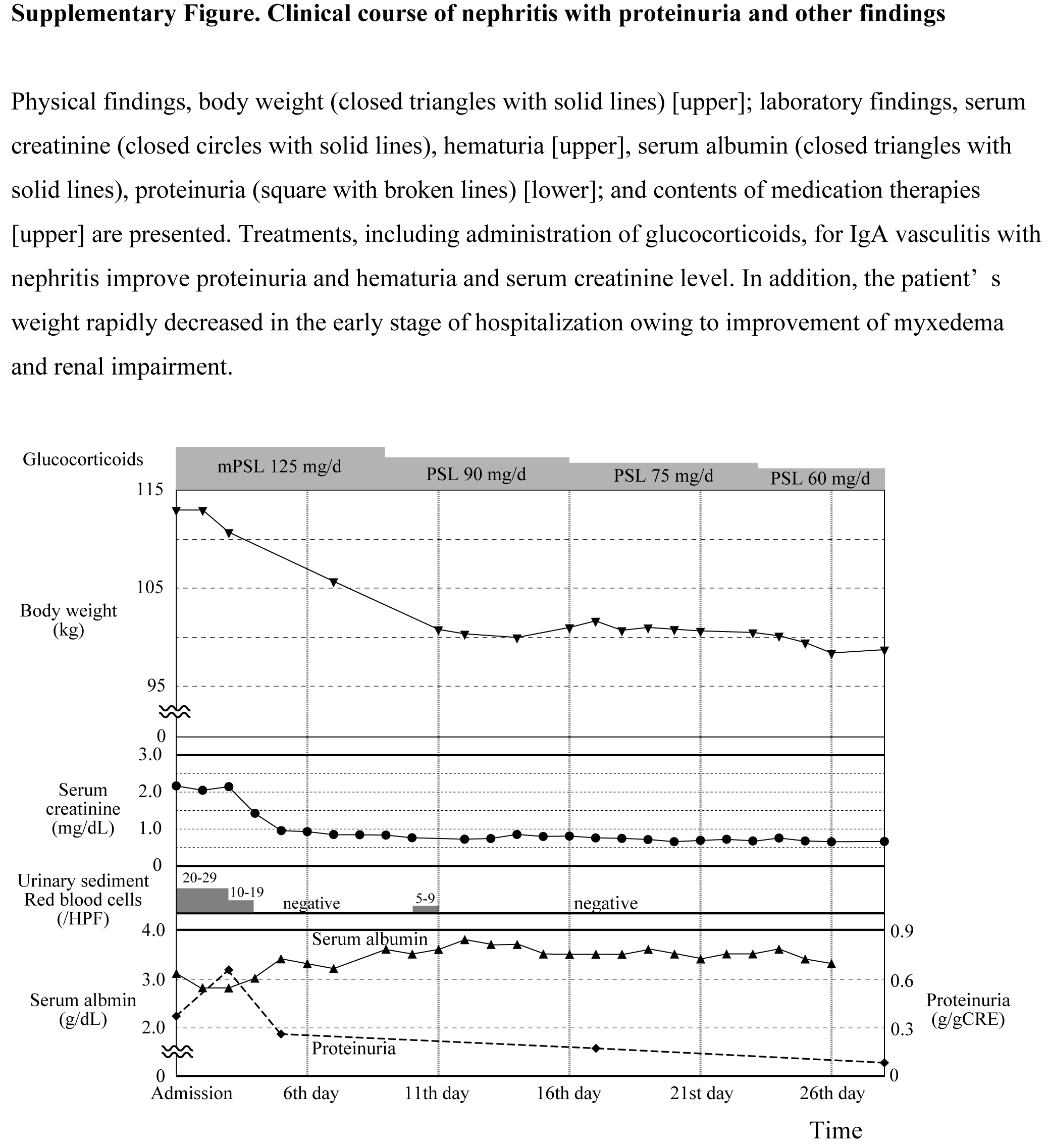

Supplement: Supplementary file 1 [file Image_1.tif]
