# Supplementary material for: Case Report: Myxedema Coma Caused by Immunoglobulin A Vasculitis in a Patient With Severe Hypothyroidism
Source: Front Immunol. 2022 Feb 18;13:838739. doi: 10.3389/fimmu.2022.838739 (PMC8895252; doi:10.3389/fimmu.2022.838739)
Supplement: Supplementary file 2 [file Table_1.pdf]

Supplementary Table. Factors known to precipitate myxedema coma

Hypothermia

Metabolic disruption

- Hypoglycemia
- Hyperglycemia
- Hypercalcemia
- Hyponatremia
- Acidosis

Infection

- Pneumonia
- Influenza
- Urinary tract infection/urosepsis
- Sepsis

Cerebrovascular accidents

- Cardiac failure
- Myocardial infarction
- Stroke

Drugs

- Amiodarone
- Beta blockers
- Diuretics
- Lithium
- Anesthesia
- Barbiturates
- Tranquilizers
- Sedatives
- Narcotics
- Phenothiazines
- Phenytoin
- Rifampin

Discontinuation of thyroxine therapy

Burns

Trauma

Gastrointestinal bleeding

Surgery

Respiratory compromise

- Hypoxemia
- Hypercapnia
